# Prevalence of *Mycobacterium avium* subspecies *paratuberculosis* IS 900 DNA in biopsy tissues from patients with Crohn’s disease: histopathological and molecular comparison with Johne’s disease in Fars province of Iran

**DOI:** 10.1186/s12879-018-3619-2

**Published:** 2019-01-07

**Authors:** Forough Zarei-Kordshouli, Bita Geramizadeh, Azizollah Khodakaram-Tafti

**Affiliations:** 1 Department of Pathology, School of Veterinary Medicine, Shiraz University, PO Box 71345-1731, Shiraz, Iran; 2 Department of Pathology, Transplant Research Center, Shiraz University of Medical Sciences, Shiraz, Iran

**Keywords:** IS900-PCR, Crohn’s disease, Johne’s disease, *Mycobacterium avium* subsp. *paratuberculosis*

## Abstract

**Background:**

Crohn’s disease is a chronic enteritis of humans that affects the gastrointestinal tract, especially the terminal ileum, cecum and colon. The etiology of this disease is still unknown but seems to be multifactorial. There are reports about the potential link between Crohn’s disease in humans and the causative agent of Johne’s disease in ruminants. Because of the prevalence of Johne’s disease in the Fars Province of Iran, the aim of this study was to investigate the prevalence of MAP in the biopsy tissues of patients affected by Crohn’s disease in this area.

**Methods:**

The study was performed from April 2015 to June 2017 at Namazi Hospital, Shiraz University of Medical Sciences, and School of Veterinary Medicine, Shiraz University, Shiraz, Iran. Intestinal biopsies of 30 patients (12 male and 18 female; mean age, 34 years; range 4–77 years) with the confirmed diagnosis of Crohn’s disease and 30 patients diagnosed as non-inflammatory bowel disease (19 male and 11 female; mean age, 38 years; range 13–68 years) were studied by molecular, histopathological and histochemical methods. Also, similar numbers of adult goats affected by Johne’s disease were studied, comparatively. DNA extractions of tissue specimens were subjected to PCR to amplify a 413-bp sequence of the IS900 gene.

**Results:**

Using IS*900*-PCR, the overall prevalence of MAP in patients affected by Crohn’s disease and non-inflammatory bowel disease were 47 and 13%, respectively. In addition, the prevalence of MAP in goats affected by Johne’s disease was 70%. Using acid-fast histochemical staining, only 7% of Crohn’s disease patients were weakly positive as paucibacillary and 43% of Johne’s disease cases were moderate to strongly positive as multibacillary. Histopathologically, granulomatous enteritis (83 and 90%), lymphoplasmacytic enteritis (17 and 14%), edema and lymphangiectasia (67 and 96%), and vasculitis (20 and 73%) were common findings in Crohn’s and Johne’s diseases, respectively.

**Conclusion:**

Our findings demonstrate a remarkable association between MAP and CD in this population, and support an etiologic relationship between MAP infection in humans and the development of CD. MAP infection in human tissue may display species-specific pathologic findings, as occurs with other zoonotic pathogens.

## Background

Crohn’s disease (CD) is an idiopathic chronic regional enteritis of human that most commonly affects terminal ileum and has the potential to affect any part of the gastrointestinal tract. The etiology of this disease is still unknown but seems to be multifactorial. The results of recent investigations have highlighted the potential link between CD in humans and *Mycobacterium avium* subspecies *paratuberculosis* or MAP [1, 2]. Millions of people all over the world are affected by CD and the rate of this disease is increasing in Iran [2]. The causative agent of Johne’s disease (JD) or paratuberculosis in ruminants is MAP which is characterized by chronic granulomatous enteritis [3]. Secretion of MAP from infected animals into their milk and excretion of MAP into their feces and therefore into the environment (feed, water, bedding and soil) make possible multiple vehicles for the transmission of MAP from infected animals to humans. MAP is able to form heat-resistant spores and persist in the host and environment [4]. MAP can survive the process of pasteurization due to its thick, waxy cell wall. MAP may lead to the development of CD in people with genetic predisposition [5]. MAP has been isolated from both raw and pasteurized milk [6–10] and from tissue biopsies and blood of Crohn’s patients [11, 12]. Even inhalation of MAP aerosols can result in infection [13]. Intestinal tissue (ileum, cecum and colon) is the most suitable site for detecting MAP bacteria. It is supposed that MAP in humans is in an obligate intracellular spheroplast form residing in the macrophages [9]. Acid-fast staining is a routine method for showing the presence of MAP in feces and tissue samples of JD [14–20]. An insertion sequence of IS900 is considered specific for MAP [21]. Therefore, IS900 polymerase chain reaction (PCR) is a rapid and routine method for MAP detection from different sources including feces, milk, intestinal tissues and mesenteric lymph nodes [22–27]. Because of the prevalence of JD in small ruminants of the Fars province of Iran, the present study was carried out to investigate the prevalence of MAP in the biopsy tissues of patients affected by CD in this area with regard to histopathological and molecular comparison with prevalent JD.

## Methods

### Study settings

The study was performed from April 2015 to June 2017 at Namazi Hospital, Shiraz University of Medical Sciences, and School of Veterinary Medicine, Shiraz University, Shiraz, Iran. We examined paraffin embedded archival tissues of 30 patients (12 male and 18 female; mean age, 34 years; range 4–77 years) with the confirmed diagnosis of CD and 30 patients diagnosed as non-inflammatory bowel disease (19 male and 11 female; mean age, 38 years; range 13–68 years) –using molecular, histopathological and histochemical methods. Also, 30 goats affected by JD and 30 healthy goat kids as control group were collected from Shiraz and Marvdasht slaughterhouses of Fars province, Iran. Ethical approval was obtained from the Ethical Committees of Shiraz University and Shiraz University of Medical Sciences. There have been no live animals used or tested on in this study. Also, we used only paraffin embedded tissues and the names and characteristics of the patients were confidential.

### Collection of samples

Intestinal biopsy samples including ileum, or cecum and colon from 30 patients with confirmed diagnosis of CD (12 male and 18 female; mean age, 34 years; range 4–77 years) and also 30 patients (19 male and 11 female; mean age, 38 years; range 13–68 years) affected by non-inflammatory bowel disease (nIBD) as the control group were selected. Thirty paraffin embedded blocks of Crohn’s patients which were diagnosed before by histopathology were selected from Namazi Hospital, Shiraz University of Medical Sciences, Shiraz, Iran. These samples were used for acid-fast staining and molecular evaluations.

Also, fresh intestinal tissues including ileum, cecum and colon from 30 goats affected by JD and 30 healthy goat kids as control group were collected from Shiraz and Marvdasht slaughterhouses of Fars province, Iran. The double samples were coded and their characteristics were recorded. For molecular method, the tissue samples were stored at − 20 °C until further analysis. For histopathological confirmation and acid-fast staining, tissue samples were fixed in 10% neutral buffered formalin, processed routinely, embedded in paraffin, sectioned at 5 μm and used for hematoxylene eosin (H&E) staining.

### Acid-fast staining method

Five μm tissue sections from paraffin blocks of all samples were used for acid-fast or Zeihl-Neelsen stain. Routinely, tissue specimens were deparaffinized and rinsed with consecutive dilutions of alcohol (96 to 70% ethanol). Slides were placed in hot Carbol fuchsin solution for 5 min. They were washed in running tap water and 1% acid alcohol until light pink and the color stopped. The slides were washed for 5 min and then rinsed in distilled water. Counterstaining was done using Methylene blue for 30 s. After rinsing in cold water, samples were allowed to dehydrate, and then cleared and coverslipped. According to a positive control slide of acid-fast stain (tissue samples of confirmed paratuberculosis) as represents mycobacteria, all fields of the stained tissue slides were observed under magnifications of × 400 and × 1000 oil immersion by light microscopy (Olympus, Tokyo, Japan). Visualization of any pink to red material with bacterial like morphology (especially inside cells) under 1000 magnification was interpreted as positive for acid-fast bacteria (AFB).

### DNA extraction

DNA was extracted from formalin fixed paraffin embedded blocks of CD samples using GeneRead™DNA Formalin Fixed Paraffin Embedded Kit (QIAGEN®) and stored at −20_°C for PCR assays.

Total genomic DNA was extracted from goat samples for PCR assays. About 2 *g* of intestinal tissue was chopped with a sterile scalpel and transferred to a 2 ml sterile microtube. Then sterile distilled water was added to the chopped tissue to 2 ml volume and centrifuged at 13000 *g* for 30 s. The supernatant layer was discarded and this stage was repeated twice. Afterward, 500 μl of lysis buffer and 50 μl of proteinase k (CinnaGene) were added and it was stirred gently. After remaining overnight at 37 °C, 500 μl of phenol-chloroform isopropanol (25:24:1) was added to the sample and it was stirred for 15 min. After centrifuging for 10 min at 10000 *g*, the supernatant layer was transferred to another sterile micro tube and the equal volume of chloroform-isopropanol (24:1) was added and it was stirred for 2 min then centrifuged at 10000 *g* for 1 min. The supernatant layer was transferred to another sterile micro tube and ethanol 100% was added 2.5 times its volume. After storing at − 70 °C for 1 h, samples were left at room temperature until thawed, then centrifuged at 10000 *g* for 1 min. The supernatant fluid was removed and the pellet was washed in 1 ml ethanol 70% followed by centrifuging at 10000 *g* for 1 min. The supernatant fluid was removed and sample was left at room temperature until total ethanol evaporated and the pellet was dry. Finally, DNA extracted pellet was resuspended in 50 μl sterile distilled water and stored at −20_°C for PCR assays.

### PCR of internal fragment of IS900

IS900 PCR was performed as described by Corti and Stephan with minor modifications [22]. DNA extractions were subjected to PCR in order to amplify a 413-bp sequence of the IS900 insertion element containing the probe target sequence. The PCR was carried out by final volume of 25 μl, containing 1× reaction buffer (CinnaGen), 5 mM MgCl_2_, 40 μM 2 deoxyribonucleoside- 5-triphosphate (dNTP; CinnaGen), 20 pmol of each primers (P90 5- GAA GGG TGT TCG GGG CCG TCG CTT AGG -3, P91 5- GGC GTT GAG GTC GAT CGC CCA CGT GAC -3) [26, 27], 0.5 U Tag DNA polymerase and 2 μl extracted DNA. Samples were amplified as follows: 94 °C for 5 min (1 cycle); 94 °C for 1 min, 59 °C for 1 min, 72 °C for 2 min (30 cycles); 72 °C for 7 min (1 cycle). The PCR products were finally electrophoresed on 1.5% safe stain agarose gel and the specific DNA fragment with the desired size of 413 bp was observed. DNA of the already identified positive case and sterilized distilled water were used as positive and negative controls, respectively.

## Results

### Histopathological findings

**Fig. 1:**
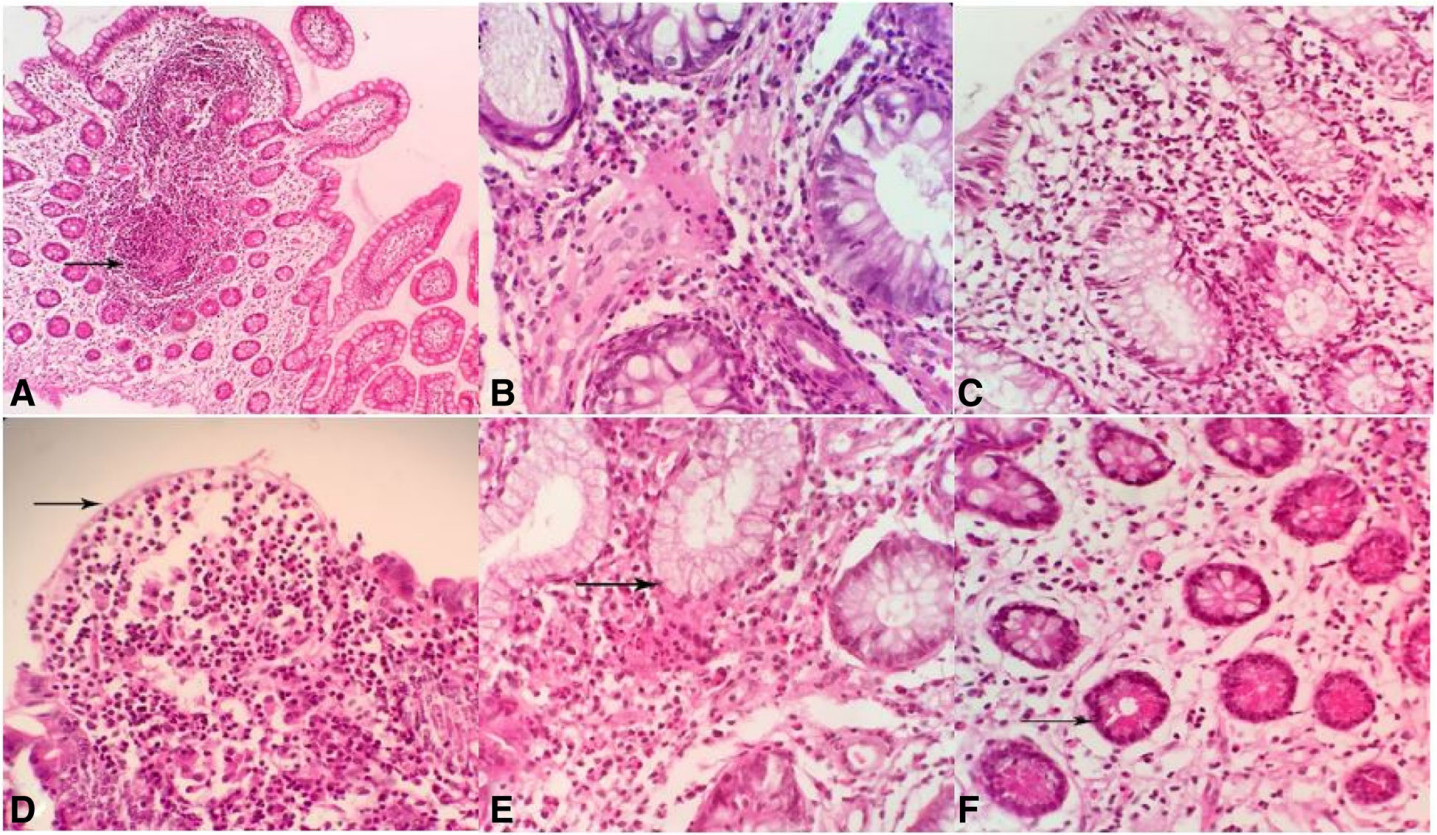
Histopathological lesions of Crohn’s disease, **a** Granulomatous ileitis: granuloma formation in the mucosa (arrow). **b** Mixed granulomatous enteritis is seen with Infiltration of lymphocytes and plasma cells in the mucosa. **c** Chronic lymphoplasmacytic enteritis: Infiltration of lymphocytes and plasma cells in the mucosa. **d** Epithelial patchy necrosis or ulceration of mucosal of ileum (arrow). **e** Pyloric gland metaplasia: compound acinar glands lined by antral type mucosa of stomach are observed in the lamina propria (arrow). **f** Paneth cell hyperplasia: These cells with numerous cytoplasmic granules are situated at the base of crypts of Liberkuhen glands (arrow) .H & E, ileum, human

The most common histopathologic lesions of CD in the present study were chronic lymphoplasmacytic enteritis, granulomatous enteritis, mucosal ulceration, pyloric gland metaplasia, lymphoid hyperplasia, Paneth cell hyperplasia, lymphangiectasia, edema and goblet cell hyperplasia. Granulomatous enteritis was observed in 83% of CD patients. Among them, 63% of biopsies were seen with noncryptolytic granulomas without central necrosis and caseation (Fig. 1a) and 20% of CD patients were observed with diffuse granulomatous enteritis (Fig. 1b) as infiltration of many lymphocytes, plasma cells and macrophages in the mucosa and submucosa of affected intestines. Lymphoplasmacytic enteritis (Fig. 1c) was diagnosed in 17% of cases of CD with infiltration of many lymphocytes and plasma cells in the mucosa and submucosa of affected intestines. Epithelial aphthoid ulcerations and mucosal microulcerations were observed in 73% of cases (Fig. 1d). Also, intraepithelial lymphocytes and neutrophils were seen in the epithelium adjacent to ulcer. Infiltration of neutrophils in the affected crypts as cryptitis and crypt abscess were also found in 40 and 33% of cases, respectively. Neuronal hyperplasia was seen in 36% cases of Crohn’s biopsies.

Increased size and diameter of lymphoid follicles in the Peyer’s patches of ileum were considered as lymphoid hyperplasia. It was seen in 33% of cases. Architectural abnormalities of the mucosa such as irregularity and blunting of the villi, atrophy of crypts and cystic dilation were also commonly found in the affected intestines. Replacement of the normal mucosal crypts of Leiberkuhn glands with antral type of stomach mucosa was diagnosed as pyloric gland metaplasia (Fig. 1e) in 40% of cases. In 17% of cases, Paneth cell hyperplasia in the ileum (Fig. 1f) was observed abnormally in the cecum and colon. These cells were situated at the base of crypts, the Leiberkuhn glands and filled with numerous prominent apical secretory acidophilic cytoplasmic granules. Lymphangiectasia and edema of mucosa and submucosa were seen as a common alteration in 67% of cases. Vasculitis in 20% and fibrinous thrombi in 30% of cases were diagnosed in mucosal and submucosal vessels. Goblet cell hyperplasia was diagnosed in 14% of cases in the ileum. Other less common findings included hypertrophy and hyperplasia of neural ganglia of the submucosa.

**Fig. 2:**
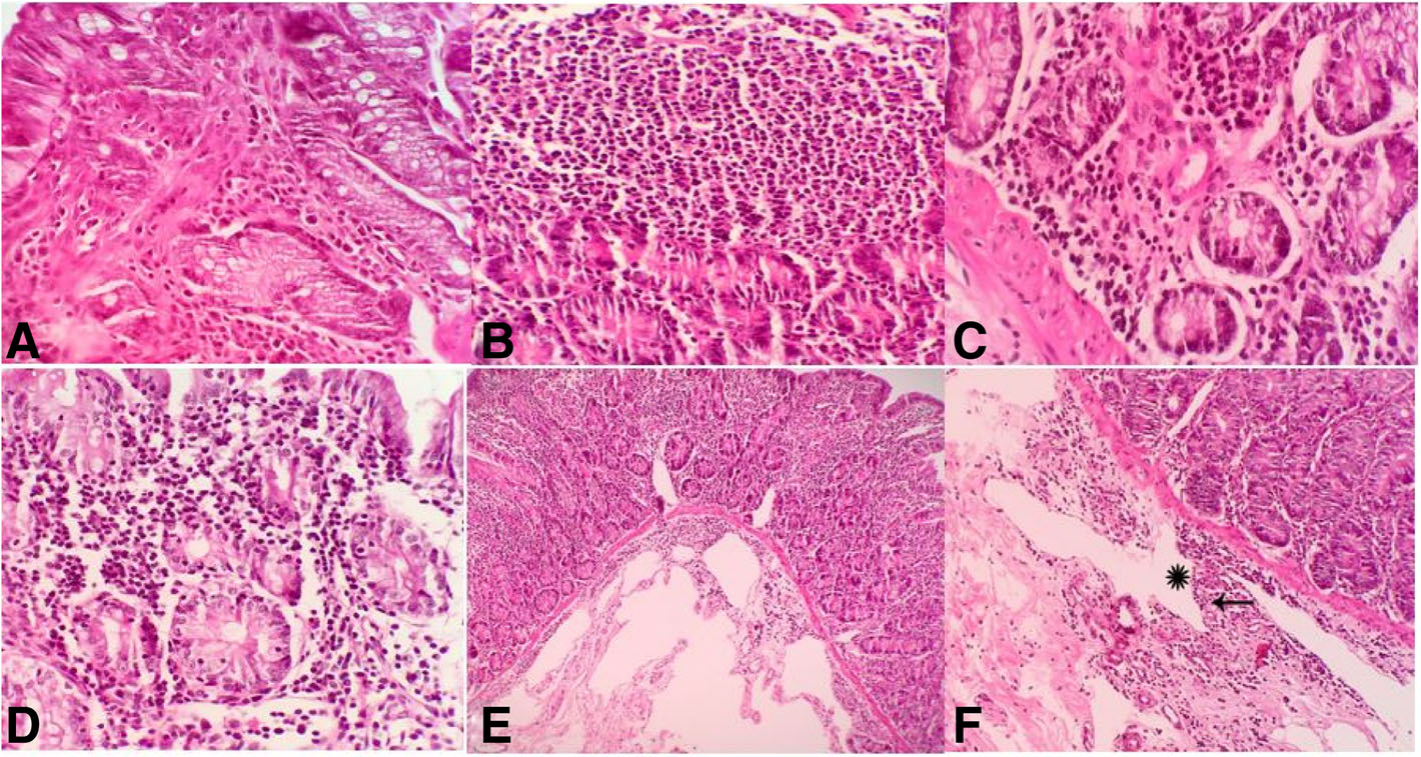
Histopathological lesions of Johne’s disease, **a** and **b** Granulomatous ileitis. Infiltration of epithelioid macrophages are seen in the mucosa. **c** Mixed granulomatous ileitis: Infiltration of epithelioid macrophages, lymphocytes and plasma cells in the mucosa and submucosa of ileum. **d** Lymphoplasmacytic enteritis. Diffuse infiltration of lymphocytes and plasma cells in the mucosa and submucosa. **e** Lymphangiectasia. Lymphatic dilation in the mucosa and submucosa. **f** Lymphangitis (starisk) and lymphocytic neuritis (arrow). H & E, ileum, goat

Most common microscopic lesions of JD were multifocal to diffuse granulomatous enteritis (multibacillary and paucibacillary forms), lymphangiectasia, edema, and lymphangitis. Granulomatous enteritis (Fig. 2a, b) was diagnosed in 90% cases of JD. Diffuse granulomatous enteritis as diffuse infiltration of epithelioid macrophages in the mucosa and submucosa of affected intestines. Diffuse mixed granulomatous enteritis (Fig. 2c) as mixed infiltration of epithelioid macrophages, lymphocytes and plasma cells in the mucosa and submucosa of affected intestines. Multinucleated giant cells were rarely seen in the affected tissues. In some villi, infiltration and aggregation of many epithelioid macrophages in the apical part make a drumstick like appearance to affected villi. By using acid-fast staining, diffuse granulomatous enteritis were divided to two forms including: multibacillary and paucibacillary lesions in 43 and 33% of cases, respectively. Also, a few of the neutrophils and eosinophils were admixed with other inflammatory cells in the lesions. Lymphoplasmacytic enteritis (Fig. 2d) was reported in 14% of JD cases as infiltration of lymphocytes and plasma cells in the mucosa and submucosa of affected intestines. Other common findings included lymphangiectasia (Fig. 2e) and edema of mucosa and submucosa in 96% and lymphangitis (Fig. 2f) in 73% of cases. Presence of lymphocytes in the submucosal neural ganglions (Fig. 2)f as lymphocytic neuritis (43%) was diagnosed. Increased size and diameter of lymphoid follicles in the Peyer’s patches of ileum were considered as lymphoid hyperplasia in 43% of cases. Atrophy of crypts associated with irregularity and blunting of the villi, were found commonly in the affected ilei. Other less common findings included reactive fibrosis in the mesenteric lymph nodes (10%), cryptitis (14%) and crypt abscess (10%). Comparative histopathological results between CD and JD are summarized in the Table 1.

**Table 1:** The comparative histopathological lesions of intestines between Crohn’s and Johne’s diseases

Lesions/diseases	CD		JD	
	N/30(%)	S	N/30(%)	S
Diffuse multibacillary granulomatous enteritis	0 (0%)	–	13 (43%)	+++
Diffuse granulomatous enteritis	6 (20%)	+	10 (33%)	++
Focal- multifocal granulomatous enteritis	19 (63%)	++	4 (14%)	+
lymphoplasmacytic enteritis	5 (17%)	+ +	3 (10%)	++
Lymphangiectasia/edema	20 (67%)	+++	29 (96%)	+++
Vasculitis/lymphangitis	6 (20%)	+	22 (73%)	+++
Lymphoid hyperplasia	10 (33%)	++	13 (43%)	++
Neuritis/neuronal hyperplasia	11 (36%)	++	13 (43%)	++
Cryptitis	12 (40%)	++	4 (14%)	+
Crypt abscesses	10 (33%)	++	3 (10%)	+
Ulceration	22 (73%)	+++	0	–
Fibrosis	16 (53%)	++	0	–
Pyloric gland metaplasia	12 (40%)	++	0	–
Thrombosis	9 (30%)	++	0	–
Paneth cell metaplasia/hyperplasia	5 (17%)	+	0	-
Goblet cell hyperplasia	4 (14%)	+	0	-

*JD* Johne’s disease, *CD* Crohn’s disease, *N* Number, *S* Severity, ^+^, mild, ^++^, moderate, ^+++^, severe

### Findings of acid-fast staining

**Fig. 3:**
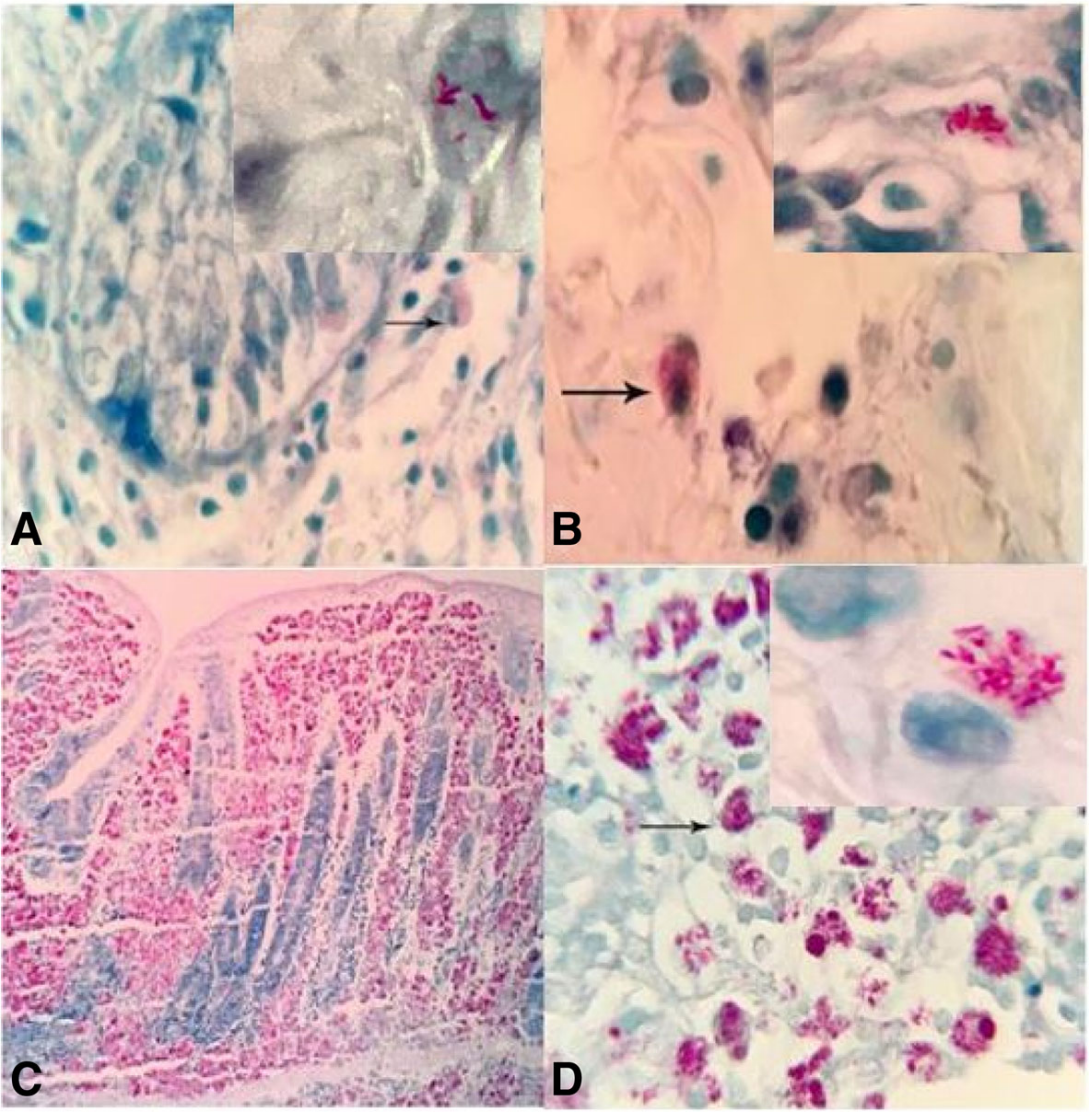
Positive acid-fast staining of affected intestines as paucibacillary in Crohn’s disease (**a** and **b**) and multibacillary in Johne’s disease (**c** and **d**). Presence of pink to red material with bacterial like morphology are seen, especially inside cells. ZN staining, human, (× 1000)

The comparative results of acid-fast histochemical staining in the CD and JD samples are shown in the Table 2. All fields of each acid fast stained slide were observed for at least 30 min with total magnifications of × 400 and × 1000 (oil immersion) by light microscopy. Weakly positive acid-fast staining as paucibacillary form of granulomatous inflammation was observed in 7% of CD (Fig. 3a, b) and moderately to strongly positive in 47% of JD cases. Multibacillary form of granulomatous inflammation was not observed in CD samples (0%) whereas 43% of JD cases (Fig. 3c, d) were positive as multibacillary lesion.

**Table 2:** Comparative results of acid fast staining and IS900 PCR

Groups Tissues	Sample Numbers(n)	Positive AFS				Positive IS900 PCR			
		Ileum	Cecum	Colon	Total	Ileum	Cecum	Colon	Total
CD	30	0%	13%	6%	7%(2/30)	50%	60%	53%	47%(14/30)
nIBD	30	0%	0%	0%	0%(0/30)	0%	3%	13%	13%(4/30)
JD	30	77%	83%	30%	90%(27/30)	63%	50%	40%	70%(21/30)
Healthy goat kids	30	0%	0%	0%	0%(0/30)	0%	0%	0%	0%(0/30)

*AFS* Acid fast staining, *CD* Crohn’s disease, *nIBD* Non-inflammatory bowel disease, *JD* Johne’s disease

### IS900 PCR findings

**Fig. 4:**
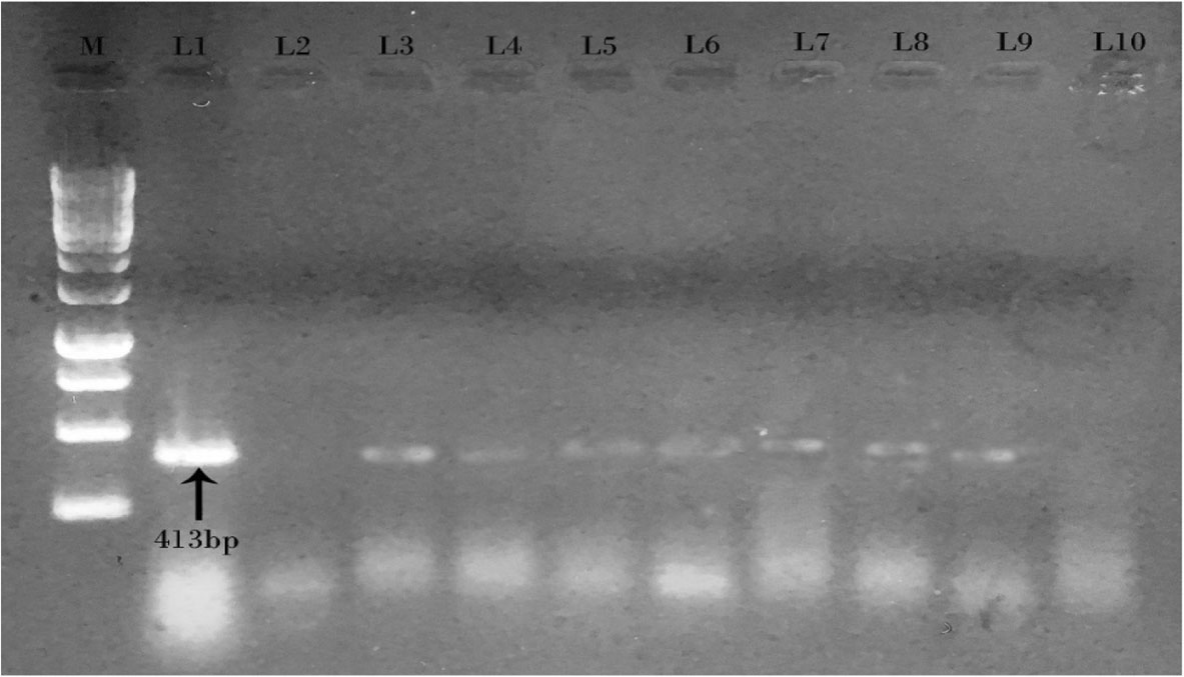
Gel electrophoresis of IS900 PCR products from Crohn’s patients. Lane M: 1 KB DNA ladder. Lane 1: Positive control MAP, Lane 2: Negative control, Lanes 3, 4, 5, 6, 7, 8 and 9: PCR products of 413 bp from Crohn’s biopsies samples, Lane 10: No product from biopsies samples

**Fig. 5:**
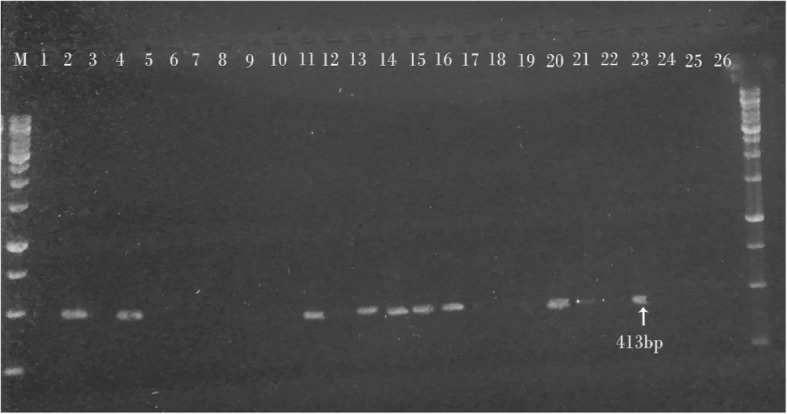
Gel electrophoresis of IS900 PCR products from intestinal tissue samples of Johne’s disease in goats. Lane M: 1 KB DNA ladder. Lane 1: Negative control, Lane 2: Positive control MAP, Lanes 4, 11,13,14,15,16,20,21 and 23: PCR product of 413 bp from tissue samples. Lanes 3,5,6,7,8,9,10,1217,18,19,25 and 26: No product from tissue samples

MAP specific IS900 gene with 413 bp was detected in 47% of CD patients (Fig. 4) and also in 70% of JD cases (Fig. 5). In the control groups, all of the samples of healthy control goat kids were PCR negative (0%), but MAP specific IS900 gene was detected in 13% of nIBD patients (Table 2).

## Discussion

The results of this study showed a relatively high prevalence (47%) of MAP DNA in biopsies of CD patients and low prevalence (13%) in control nIBD patients in Fars province of Iran. Zamani et al. (2017) reported positivity for IS900 PCR in 64% of CD, 33% of ulcerative colitis (UC) and 9.7% of control nIBD samples from patients biopsies in Tehran province of Iran [2]. Speculation has existed for many years. JD is zoonotic and somehow causative of CD in human beings [3, 28]. CD as one of the main types of inflammatory bowel disease (IBD) is described as a relapsing disorder with high morbidity and uncertain pathogenesis that can occur from early childhood to late adulthood. Although the etiology of CD is largely complex and unknown, it is thought to be caused by a combination of genetic and environmental factors that affect the immune responses. Among environmental factors**,** MAP seems to have the important role in the pathogenesis of CD, despite conflicting reports. Some researchers showed the involvement of MAP in CD [2, 15, 24, 26, 29] whereas a number of reports could not confirm such association [6, 30–32]. In genetically susceptible individuals cell-mediated immune responses to intestinal bacteria seems to play an important role in its etiology.

In different provinces of Iran, prevalence of JD and presence of MAP in farms of small or large ruminants have been reported [33–35]. Therefore, during subclinical or clinical stages of JD, MAP is shed through feces and milk which can contaminate the environment and dairy products. The nature and pathology of CD in human is somehow similar to JD in ruminants. In the present study, similar histopathological changes including granulomatous enteritis, lymphoplasmacytic enteritis, edema, lymphangiectasia, fibrosis, vasculitis, cryptitis and crypt abscess were observed in both CD and JD. Also, other reported diagnostic features of CD such as patchy necrosis of epithelium, Paneth cell hyperplasia, nerve fiber hyperplasia, and architectural distortion associated with increased intraepithelial lymphocytes were in accordance with our results [36–40]. In spite of remarkable histopathologic similarities between CD and JD in our study, specific pathologic findings of CD patients (that were not found in JD) include ulceration, fibrosis, goblet cell hyperplasia, Paneth cell hyperplasia, thrombosis, nerve fiber hyperplasia, and architectural distortion. Although, there is no discussion in the veterinary literature of MAP infection causing these pathologic findings, there are some reports such as goblet cell hyperplasia of ileum in acute and chronic MAP infection in cattle [41, 42], thrombosis of lymphatic vessels in ileum of deer JD [43], multiple foci of ulceration in sheep JD [44]. MAP infection in human tissue may display species-specific pathologic findings, as occurs with other zoonotic pathogens. Van Kruiningen (1999) in a review report, concluded that presence of a little or superficial pathologic similarity between CD in humans and JD of animals, provides no support for association between MAP and CD [45].

In the present study, fibrosis of intestinal mucosa was found in 53% of CD patients and in 10% of mesenteric lymph nodes of JD cases. In agreement with our study, reactive fibrosis of affected mesenteric lymph nodes was reported in JD deer and sheep [44, 46]. Crypt abscesses as focal aggregation of neutrophils within the intestinal crypts was diagnosed in 33 and 10% of CD and JD cases, respectively. This lesion was reported by other studies in cattle and sheep affected by paratuberculosis [41, 46], and also in non-human primates MAP infection [47].

Infiltration of lymphocytes and macrophages in the mucosa and submucosa, crypts atrophy, lymphoid hyperplasia and the presence of microgranulomas in the mucosa of affected intestines were observed similarly in both CD and JD and are in agreement with other reports [18, 19, 48, 49]. In the present study, using acid-fast histochemical staining, only 7% of CD patients were weakly positive as paucibacillary and 43% of JD cases were moderate to strongly positive as multibacillary. Our findings about paucibacillary and multibacillary forms of granulomatous enteritis in JD are in agreement with previous reports [48, 49]. There are not many MAP organisms in the tissues of CD patients and only small quantities of the microorganism may be present. In these tissues, MAP occur in a round, coccoid form that stains with acid fast stains. Detection of MAP in tissues requires oil immersion microscopy and microscopic detection limit is governed more by bacterial burden than by staining method [17, 50]. In the JD cases with multibacillary lesions, mucosal atrophy and replacement of epithelioid macrophages instead of intestinal glands have been reported in other studies [51–53]. It has been suggested that paucibacillary and multibacillary lesions are associated with strong cell mediated and humoral immune responses, respectively [54, 55].

In this study, infiltration of neutrophils was more common in CD lesions than JD. Marks et al. (2006) described when the numbers of functional neutrophils are not enough for the effective clearance of bacteria; bacteria will be phagocytized by macrophages to make the granuloma and chronic inflammation typical of CD [56]. Similar to JD, granulomas without central caseous necrosis which were observed in the lamina propria of affected intestines in CD patients of our studies are in agreement with others [37, 53, 57]. These granulomas are not cryptolytic or dependent on crypt injury [56] but cryptolytic granulomas in ulcerative colitis patients was reported [58].

In our study, crypt irregularity was observed in CD biopsies. Crypt irregularity is characterized by crypt distortion (non-parallel crypts, variable diameter or cystically dilated crypts), crypt branching and crypt shortening [40]. Tanaka et al. (1999) have reported that crypts should be regarded as abnormal if more than two branched crypts exist in a well-orientated biopsy specimen [58]. While focal architectural abnormalities favor CD, pseudovillous appearance of the colorectal surface, crypt irregularity, and reduced crypt numbers and crypt epithelial polymorphs is proposed more consistent with a diagnosis of ulcerative colitis [59, 60].

In the present study, unlike JD, pyloric gland metaplasia was diagnosed in 40% of CD cases. It is referred as pseudopyloric gland metaplasia or glandular mucoid metaplasia that is a feature of chronic mucosal inflammation and related to mucosal ulceration and repair [38, 61]. In our study, Paneth cell metaplasia was diagnosed in 17% of cecal biopsies of CD patients. Tanaka et al. found a correlation between Paneth cell metaplasia and crypt architectural distortion of chronic IBD [62]. It is reported that Paneth cell numbers were significantly increased in IBD at all sites except the rectosigmoid in ulcerative colitis and the caecum and rectum in CD [63].

Nancy and Buckley (2008) stated that reports of significant number of positive MAP CD patients revealed that an association between MAP and CD does exist [29]. Unlike ruminants paratuberculosis, CD is not a lepromatoid disease with an abundance of MAP laden macrophages. Also, in initial stage of MAP infection, no macroscopic and histological lesions including acid-fast organism can be detected [64]. This study showed, unlike in JD cases, most of the CD intestinal lesions (93%) were acid-fast negative. Acid-fast stain is necessary for demonstrating the mycobacterial bacilli. Detection of one or more mycobacteria in an area of granulomatous inflammation is highly specific and indicative of infection. It is believed that more than 10 mycobacteria per milligram of tissue are usually necessary to detect the organism by light microscopy, so a negative stain does not exclude a diagnosis of mycobacterial infection [65]. This level of MAP in the tissue is needed in order for histochemical stains to show the organism, and there are not many MAP organisms in the tissue, because of the strong cell wall mediated immune response. In the present study, the acid-fast bacilli were detected in most of the JD intestinal lesions. This finding is in agreement with other investigations [7, 16–18]. Only small quantities of the microorganism are present in the tissues of CD [20]. The absence or sparcing of MAP in the mucosal lesions is related to strong cell-mediated immune response with poor or absent humoral response. The intracellular phenotype of MAP in Crohn’s patients does not illustrate an acid-fast-positive lipid rich mycobacterial cell wall. MAP in its protease resistant acid-fast negative phenotype parasitized immunoregulatory macrophage and other cells. It is associated with a variable immune dysregulation [50]. This matter describes why histological examinations cannot detect acid-fast cells in tissues of CD patients [28]. Paucibacillary form infections revealed that mycobacteria could only be visualized by careful examination under 1000 oil immersion [66].

Unlike CD, MAP in JD samples has intact cell walls and abundant acid-fast bacilli can be observed in multibacillary lesions. Hence, infected ruminants with JD, shed numerous MAP from feces and increase the risk of its transmission to humans. Bharathy et al. have detected MAP DNA in milk samples of goats [10]. This organism is a resistant bacterium to pasteurization temperature for having thick lipid cell wall [66, 67]. Consumption of milk and different dairy products like cheese can increase the risk of transmission to human [10, 21–23, 33, 67].

Several studies have revealed MAP DNA in intestinal biopsies, peripheral blood and breast milk samples of CD patients [5, 14, 30, 31, 68]. There are reports about confirmation of contamination of water sources, aerosols, dairy products, river waters, and even domestic tap water and survival from chlorine disinfection of MAP [69, 70]. The isolation and detection of MAP in breast milk of CD patients supports the possibility that it can be a systemic infection in humans [68]. It results in activation of immune system to development of inflammatory bowel disease [71].

In this study, despite the presence granulomatous lesions, MAP could not be detected by PCR in a number of CD patients. The absence of MAP DNA in the majority of our Crohn’s paraffin blocks may be explained by a strong cell-mediated immune response to MAP in humans with CD as discussed above, which results in paucibacillary lesions. Collins et al. (2000) used different methods including IS900 PCR to detect MAP in IBD patients and in controls and found IS900 was positive significantly in CD patients (19.0%) and UC patients (26.2%) than from controls (6.3%). The complex interplay of potential age-dependent effects, host genetic effects, immune responses, immunosuppressive drug therapy effects, BCG vaccination, rate of exposure to MAP or other risk factors on infection susceptibility to MAP must be taken into consideration in future studies [72]. The effect of anti-mycobacterial therapy on CD is unclear [73]. Also, McNees et al. (2015) expressed that MAP is widespread in dairy cattle and because of resistance to pasteurization and chlorination, humans are frequently exposed through contamination of food and water [74].

Also, PCR amplification can create false-negative results [75]. False-negative results (the specific error at issue) can be caused by a low number of MAP targets and/or PCR inhibitors.

Schwartz et al. (2000) described a higher frequency of MAP in CD patients (37%) than healthy controls (5.6%). They found MAP DNA at a higher percentage (86%) in surgically resected tissue samples in comparison to tissue biopsies (20%) and suggested that MAP may be located below the mucosal layer than on the apical surface area [31]. Therefore, larger specimens that contained the deeper portions of the bowel wall are more apt to yield positive results.

Our study supports the possible role of MAP in triggering CD, whereas low IS900 PCR detection rates were observed in the control nIBD group. Similar to results of this study, some researchers have obtained low prevalence of MAP in the intestinal tissues of nIBD patients [20, 76–78]. No study has done a long-term follow up on such persons to see if they remain healthy or are in the early stage of a disease induced by MAP. This could be CD or any number of other diseases linked with MAP such as sarcoidosis, Blau syndrome, type 1 diabetes, Hashimoto thyroiditis, and multiple sclerosis [79].

## Conclusion

Our findings demonstrate a remarkable association between MAP and CD in this population, and support an etiologic relationship between MAP infection in humans and the development of CD. MAP infection in human tissue may display species-specific pathologic findings, as occurs with other zoonotic pathogens.

## Abbreviations

AFS: Acid-fast staining
CD: Crohn’s disease
IBD: Inflammatory bowel disease
JD: Johne’s disease
MAP: *Mycobacterium avium* subspecies *paratuberculosis*
nIBD: Non-inflammatory bowel disease
PCR: Polymerase chain reaction
UC: Ulcerative colitis
